# Annuloplasty for mitral valve repair in degenerative disease: to be flexible or to be rigid? That’s still the question

**DOI:** 10.1007/s12055-020-01001-3

**Published:** 2020-09-18

**Authors:** Vito Domenico Bruno, Ettorino Di Tommaso, Raimondo Ascione

**Affiliations:** Translational Health Science, Faculty of Health Science, Bristol Heart Institute, University of Bristol Medical School, Marlborough Street, Bristol, BS2 8HW UK

**Keywords:** Mitral valve repair, Mitral valve regurgitation, Mitral ring, Annuloplasty

## Abstract

The choice of ring for mitral valve repair is still largely left to the surgeon's preferences and there are no specific guidelines regulating this decision. Despite this previous researches have described important features appertaining to each of the different types of rings currently available. Particularly, the debate is still open in regards to the flexibility that these devices should or should not have. Later in this issue of the Journal, Panicker and colleagues have reported their results with flexible and rigid rings in mitral valve repair. The results are very interesting and once again are highlighting the importance of using the right ring for the right disease.

Mitral valve (MV) repair surgery is the gold standard for the treatment of severe mitral valve regurgitation in patients with degenerative valve disease [1, 2] and annuloplasty techniques are a key part of an effective and long-lasting repair [3]. To this end, there are several types of rings available with differences in materials used, 2D and 3D shape, complete or semi-complete, rigid, semi-rigid, or flexible. For simplicity, these are often referred to as rigid or flexible rings [3]. The rationale for using flexible rings in degenerative MV disease is based on the preservation of the dynamic systolic-diastolic motion of the mitral valve annulus and its role in the contractile performance of the left ventricle (LV). Its use vs rigid rings to treat degenerative MV disease has been long debated with some of the most interesting studies pooled in a meta-analysis including 4 randomized trials and 8 case-control studies [4]. This showed that the impact of the flexible annuloplasty was comparable with the rigid ring in terms of in-hospital mortality, need for reoperation, recurrent significant mitral regurgitation (MR), late survival, shortening fraction, and LV volumes and diameters. However, the flexible annuloplasty was associated with higher LV ejection fraction, larger residual mitral valve area, and reduced peak velocity across the repaired valves, which are all important dynamic and functional differences [4]. These findings are in keeping with the outcome of translational research suggesting that the use of flexible rings preserves the posterior annular movements [5]. In a large animal experimental model, Yokote and colleagues have evidently shown that the antero-posterior diameter of the MV annulus is significantly reduced after the implantation of a ring, and this effect is more pronounced with the rigid rings, while the transverse diameter seems to be more affected by the flexible ring [5]. Moreover, this preclinical study confirmed that the rigid ring restricts the annular mobility and the contraction ratio of the MV annulus during the cardiac cycle [5].

Noticeably, it must be said that the use of flexible rings is justified only for degenerative MV disease and not in patients afflicted by ischaemic or functional MV disease. Indeed, in patients with ischaemic congestive heart failure, the use of rigid, complete, and downsizing rings has been associated with reduced likelihood of developing long-term recurrent MR requiring reoperation when compared with flexible and semi-complete rings [6].

It must be emphasized that despite these findings and the available evidence from clinical and translational studies and guidelines, the selection of which ring to use is not regulated and is still typically dictated by surgeon’s preference. In the latest issue of the Journal, the paper by Panicker and colleagues [7] provides a valuable and pragmatic contribution to the debate on the appropriateness of using flexible or rigid rings to treat degenerative MV regurgitation. In a retrospective analysis, the authors have followed up 112 young patients undergoing MV repair for primary degenerative MV regurgitation to ascertain the mid-term impact of flexible and rigid rings in terms of recurrent MV regurgitation and LV positive remodelling. The study suggests that both rings are safe and effective in treating MV regurgitation with comparable and acceptable 5-year outcome, in keeping with the suggestions from previous studies [3]. One remarkable finding of this study is the reduction in LV size at 5 years in both groups with a trend favouring the flexible ring, although not significant [7], also in keeping with the outcome of the previously cited meta-analysis [4]. More than 30 years ago, Spence and colleagues [8] were already able to compare the effects of rigid and flexible rings in isolated porcine hearts, and found that fixation of the mitral annulus was disadvantageous to the systolic function of the left ventricle. Other clinical reports have already highlighted the beneficial effect of a flexible ring on LV function [9, 10], although the latest prospective randomized trial has failed to confirm a clinical advantage of the flexible ring in terms of LV function and size [3].

In Panicker’s study, with on-table echocardiography showing no residual MR in both groups, it is interesting to see that the recurrence of ++/+++ MR at 5 years was 10/42 (23.1%) vs 11/70 (15.7%) in the rigid group vs flexible group, respectively. These interesting results for the flexible rings are in keeping with previously reported data [11] and once again support the reliability of the flexible rings in the context of degenerative MV disease. Kanemitsu et al. have shown a freedom from recurrence of significant MR after flexible ring of 92.5 ± 2.2% at 10 years and 73.1 ± 7.1% at 15 years. Panicker and colleagues are confirming the durability of these repairs suggesting a possible better late dynamic performance of flexible rings, although this important finding is not highlighted enough in the paper. In terms of complications, the current study reports a single case of early haemolysis triggering reoperation, although it is not obvious in which group: the authors should be congratulated for this very small rate of complications. On the other end, a limitation of the study is the variety of ring models used for both types of rings over a total population of 112 patients. This variability might reflect the fact that different surgeons have contributed patients to this series. In a sense, using a wide range of rings provides an opportunity to undertake subgroup analysis among flexible and rigid rings, something that in this study was not advisable due to the relatively small overall sample size. Additional limitations might be the relatively small sample size and the lack of data on residual gradients across the valve early or at 5 years post-surgery.

Perhaps, another omitted consideration is related to the use of pericardial rings: if we focus on flexibility as well as biocompatibility, pericardial rings may be regarded probably the most flexible of all and have been associated with promising results [12, 13]; some authors consider them as a more physiologic correction that preserves mitral annulus motion [14], with potential to be less thrombogenic and prone to infection, although this remains still to be demonstrated.

There is an important final consideration to make on this exciting field of MV repair surgery: over and above the type of ring being used or the techniques of leaflet repair adopted, a key determinant of early and late success of MV repair is the surgeon’s expertise. There is now strong data suggesting that the outcome of MV repair is superior when done by high-volume surgeons in centres of MV repair excellence [15]. This applies not only to MV repair rates but also freedom from reoperation and survival [15]. Indeed, evidence suggests that in expert hands, the MV repair success rate should be > 80–90%, with in-hospital survival of > 99% in healthy patients. It is on these grounds that MV repair surgery for degenerative MV disease is now indicated also in asymptomatic patients with severe MR in keeping with the latest guidelines [3, 4].

In conclusion, the authors have contributed a valuable study from India providing additional information to this global debate, for which they should be congratulated. In the meantime, while the debate on flexible vs rigid rings for degenerative mitral valve disease continues to progress, it should be reiterated here that MV repair for degenerative disease should be done by expert surgeons to ensure very high MV repair success rates and excellent early and long-term outcomes. Hence, surgeon and centres should invest and specialize in this art, providing a surgical practice by high volume and by expertise to include also excellent on-table echocardiography, dedicated annual audits, and MV out-patient clinic services to ensure the best early and long-term outcome for our patients.
